# Assessment of Geant4 Prompt-Gamma Emission Yields in the Context of Proton Therapy Monitoring

**DOI:** 10.3389/fonc.2016.00010

**Published:** 2016-01-28

**Authors:** Marco Pinto, Denis Dauvergne, Nicolas Freud, Jochen Krimmer, Jean M. Létang, Etienne Testa

**Affiliations:** ^1^CNRS/IN2P3 UMR 5822, IPNL, Université de Lyon, Université Lyon 1, Villeurbanne, France; ^2^CREATIS, CNRS UMR 5220, INSERM U1044, INSA-Lyon, Centre Léon Bérard, Université de Lyon, Université Lyon 1, Lyon, France

**Keywords:** proton therapy, hadrontherapy, prompt gammas, Geant4, online monitoring, in-beam monitoring, collimated camera, nuclear fragmentation models

## Abstract

Monte Carlo tools have been long used to assist the research and development of solutions for proton therapy monitoring. The present work focuses on the prompt-gamma emission yields by comparing experimental data with the outcomes of the current version of Geant4 using all applicable proton inelastic models. For the case in study and using the binary cascade model, it was found that Geant4 overestimates the prompt-gamma emission yields by 40.2 ± 0.3%, even though it predicts the prompt-gamma profile length of the experimental profile accurately. In addition, the default implementations of all proton inelastic models show an overestimation in the number of prompt gammas emitted. Finally, a set of built-in options and physically sound Geant4 source code changes have been tested in order to try to improve the discrepancy observed. A satisfactory agreement was found when using the QMD model with a wave packet width equal to 1.3 fm^2^.

## 1 Introduction

Particle therapy, namely proton and carbon-ion therapy, has been the subject of growing interest, primarily due to the favorable ballistic properties of ion–matter interactions, which allow for a high degree of dose conformality in the tumor while minimizing the dose in the healthy tissue. However, such properties pose some challenges in terms of quality assurance of the treatment when compared, for example, to photon radiation therapy since ions are more sensitive to both planning and treatment uncertainties (1, 2). Several verification protocols and monitoring approaches have been proposed to address this issue, among which the detection of prompt gammas (PG) for ion range monitoring. Prompt gammas are the result of nuclear interactions between the incident ion and the tissue nuclei. Their emission can be considered as instantaneous after the interaction, thus providing a strong correlation with the ion range (3, 4). Moreover, when compared with the positron emission tomography monitoring, already in clinical use for the same purpose, prompt-gamma monitoring does not suffer from signal washout and time dependency. In addition, the energy threshold for the nuclear reactions producing positron emitters is higher than the one for the emission of PG, hence a better correlation with the ion range is observed for the latter (5). However, the need for dedicated devices with high acquisition rate capabilities, the broad energy range of the emitted prompt gammas, and the extensive background render it a particularly demanding technique.

The inherent complexity of the nuclear processes leading to the emission of prompt gammas makes Monte Carlo tools one of the main resources employed in the study of this form of particle therapy monitoring, namely in terms of camera optimization [e.g., Ref. (6–10)]. In this regard, Geant4 (11) has been one of the chosen tools due to ease of use and open-source distribution. However, as already described in the literature (8, 12–15), the hadronic inelastic models implemented in Geant4 tend to overestimate prompt-gamma emission. There is no evidence so far that the spatial prediction is also affected; hence, it is still possible to use the spatial prompt-gamma distributions to find correlations with ion range. Nevertheless, relying on an overestimated signal raises a concern for the optimization of devices to exploit the information provided by the prompt-gamma emission since the precision to detect ion range shifts is inversely proportional to the collected signal (16).

The present study addresses the issue of discrepancies in prompt-gamma emission yields after proton irradiation using Geant4 by comparing all applicable proton inelastic models with experimental data. In addition, we propose and test several approaches within the existing models to try to improve the accuracy of Geant4 for prompt-gamma emission yields.

## 2 Materials and Methods

### 2.1 Experimental Data

The experimental data were collected during an experimental campaign at the Westdeutsches Protonentherapiezentrum Essen (WPE, Essen, Germany) (17). The setups comprised a single-slit collimator, a detector, and a target aligned with the beam axis. The single-slit collimator was positioned orthogonally with respect to the beam axis and the alignment and positioning of the different setup elements were accomplished by means of lasers and rulers, respectively. The cylindrical polymethyl methacrylate (PMMA) target with a 75-mm radius and 200-mm length was positioned on top of a moving table, thus allowing to perform measurements along the target. The step size of the different longitudinal positions was not fixed, and it was dependent on factors like ion range and need for a better description of some prompt-gamma profile features (e.g., at the target entrance or close to the end of the ion path). The collimator was made of a tungsten alloy with a 4-mm slit opening and, when applicable, the shielding consisted of lead blocks. Two setups were considered. The data of the first setup (setup 1) were collected by means of a LYSO detector, while in the second one (setup 2), the LYSO and LaBr_3_ detectors were used.

**Figure 1:**
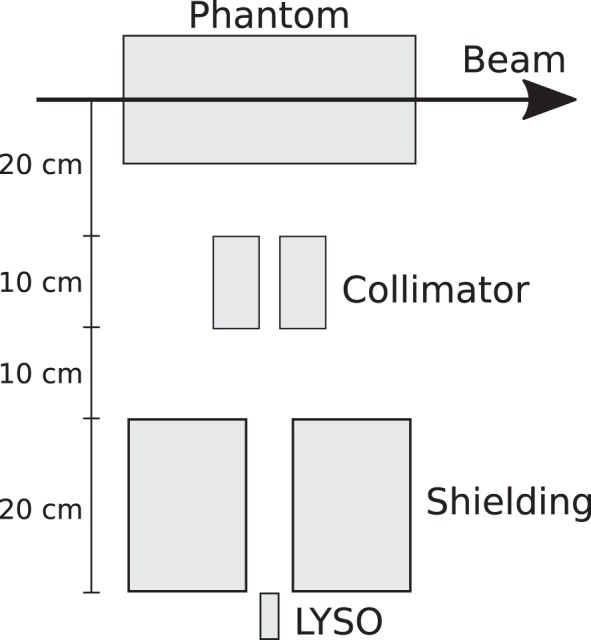
**Schematic illustration of setup 1 (not to scale)**.

**Figure 2:**
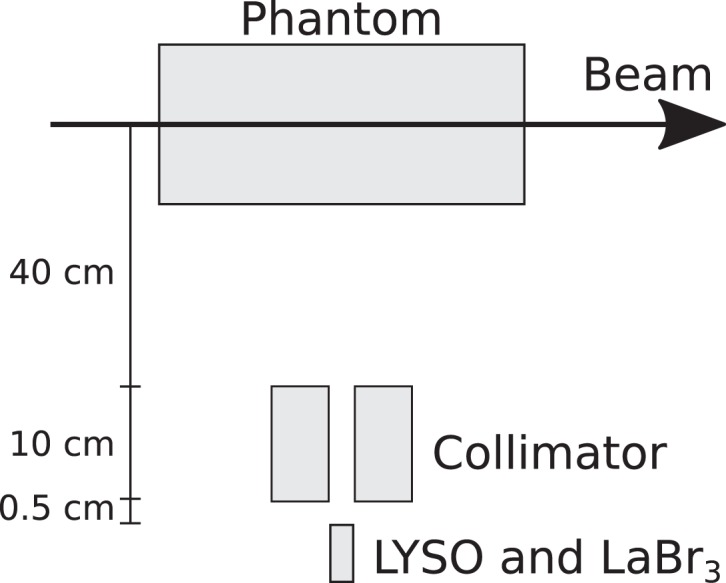
**Schematic illustration of setup 2 (not to scale)**.

A schema of setups 1 and 2 can be observed in Figures 1 and 2, respectively.

In order to select the prompt gammas from the extensive background, the time-of-flight (TOF) technique was used in conjunction with a VME-based acquisition system with NIM modules and discrete logic and analogic electronics. The TOF windows applied during the analysis were always sufficiently large to include all the visible prompt-gamma events. The TOF stop signal was given by the high-frequency (HF) signal of the cyclotron running in pulsed mode. The stop signal was actually provided by a discriminator converting the HF signal into a digital logic one whose frequency was divided by a factor of ~5 with respect to the HF frequency to cope with the time-to-amplitude (TAC) module limitations. The TOF spectra measured in these conditions correspond therefore to ~5 periods of the HF signal. The circular beam spot was around 5 mm sigma at isocenter, considering a Gaussian spatial beam distribution (18). Energy thresholds were also applied to the data in order to reduce the background component. The energies considered were obtained after calibration with gamma sources. Therefore, it is an absorbed gamma-equivalent energy but, for the sake of simplicity, it will be simply referred to as energy. The lower-energy threshold for the detectors in the post-processing steps was 1 MeV, while the upper one was 7 and 12 MeV for the LYSO and LaBr_3_ detectors, respectively. The difference between the two upper-energy thresholds is due to the distinct usable energy range of each detector. In addition, scalers were also used to account for the dead time of the acquisition system.

This experiment was conducted using a single proton energy (160 MeV) and with a suitable beam intensity to avoid pile-up and excessive dead time. The number of incoming protons was given by the ionization chamber (IC) placed inside the beam nozzle, thus allowing for the normalization of the data. The IC was calibrated against a Bragg peak chamber positioned at the target entrance.

In order to make a better comparison between experimental and simulated data, both data sets are subjected to a background subtraction procedure. It was decided to follow the procedure followed by Pinto et al. (17), where the TSpectrum routine of ROOT (19) is used.

Additional details about these experimental data can be found elsewhere (17), namely, in terms of TOF analysis and absolute yields.

### 2.2 Geant4 Data

The Geant4 version 10.01.p02 was used as it was the last stable release at the time of the present study. In this version, there are five proton hadronic inelastic models for the energy range considered herein: binary cascade (BIC), Bertini cascade (BERT), precompound (PRECO), Liège intranuclear cascade (INCL), and quantum molecular dynamics (QMD). It should be noted that the QMD model is usually never considered for proton interactions, but its implementation in Geant4 is fully prepared for using it in such a case. QMD is the most comprehensive hadronic inelastic model in Geant4 and its complexity is often regarded as needless to describe proton interactions since there are other models able to perform the same task with similar accuracy but requiring much less computing time (usually around one order of magnitude less).

The description of these models is outside the scope of the present paper, but additional information can be found in the Geant4 web page (https://cern.ch/geant4) and the references therein.

The simulation of the experimental setups requires a high amount of computing resources due to its small solid angle; hence, a method to consider all possible models was selected. First, a proton inelastic model was selected to be used for the simulation of the experimental setups and subsequent comparison with the experimental data. Since the developers of Geant4 recommend the use of the BIC model for the present case, it was decided to choose it to be the reference model. In a second stage, the physical models were used to retrieve a longitudinal distribution of prompt gammas escaping the target with an angular acceptance of ±1.5°[similar to the procedure followed by Biegun et al. (20)]. Finally, the comparison between the experimental data and those from the simulation of the full setups with the reference model provides an estimate of the corresponding experimental yields for the case where PG escapes from the target. This makes possible and a meaningful comparison between all the physical models while optimizing the use of computing resources. Nevertheless, such an approach discards the potential influence of the neutron-induced gammas created in the collimator and/or shielding. In any case, those events should not overlap with the PG peak and after background subtraction and TOF selection they are assumed not to have an impact on the results (17).

The implementation of the experimental setups in Geant4 included target, collimator, shielding blocks, detectors, and nozzle components. In order to account for the extensive background due to the pile-up of events from previous proton bunches, an off-line procedure was applied to the simulated data to mimic the beam frequency (106 MHz). Additionally, as mentioned previously, the simulated data were analyzed with the same software as the experimental ones, thus reducing possible discrepancies that could have been introduced by the use of different analysis routines. It should be noted that the same experimental absorbed energy thresholds were used for the simulated data (i.e., 1–7 MeV for the LYSO detector and 1–12 MeV for the LaBr_3_ one).

Table 1 shows the most relevant physical models other than the proton hadronic inelastic ones used in the simulations.

**Table 1:** **The most relevant physical models used for the simulations (not including the proton inelastic ones)**.

Hadronic inelastic neutrons		Hadronic inelastic ions (heavier than H^*+*^)	Others
*<*20 MeV	*≥*20 MeV	
G4NeutronHPInelastic	G4BinaryCascade	G4IonBinaryCascadePhysics	G4HadronElasticPhysicsHP

### 2.3 Comparison between Experimental and Simulated Data

**Figure 3:**
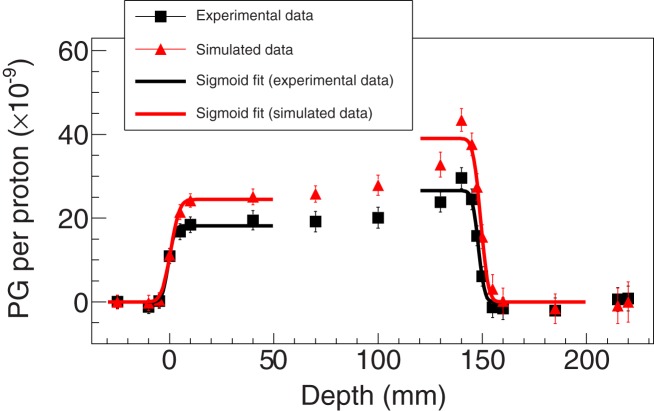
**Experimental and simulated data for setup 1 using the LYSO detector and considering an energy selection of 1 ≤ energy ≤ 7 MeV**. The fits using sigmoid functions in order to retrieve the PGPL are also shown (the range plotted is the same of the fit procedure). The simulated data were obtained with the BIC model for proton inelastic interactions.

The Geant4 benchmarking included two endpoints, arguably the two most relevant ones: yields and information correlated with the ion range. The former has an impact on, for example, camera optimization, while the latter plays a major role in a monitoring scenario. The yields were assessed using the reference model and comparing its outcomes with the experimental data. The discrepancy was then evaluated by computing the average relative difference between selected simulated and experimental data points. The rationale for such a selection was the need to avoid high-gradient signal regions since they could have a significant and misleading impact on the calculation of the relative difference due to spatial uncertainties. Therefore, the points considered were between 20 mm (to avoid the entrance of the target positioned at 0 mm – see, e.g., Figure 3) and 140 mm (to avoid the PG profile falloff). The projected proton range for the experimental data was 154.72 mm (21) (not including nozzle elements).

Concerning the information provided by the prompt-gamma profile correlated with the proton range, Pinto et al. (22) proposed the use of the quantity designated as prompt-gamma profile length (PGPL) to measure the distance between the rise in the prompt-gamma profile at the entrance of a target or patient and the falloff close to the end of the ion path. They showed that this quantity is correlated with the ion range for the case of carbon-ion irradiation. Herein, the same approach will be used, and it comprises the fit of sigmoid functions to both the prompt-gamma profile entrance and falloff. The PGPL is obtained through the subtraction between the two inflection points retrieved after the fit to both positions. This function has been initially proposed by Henriquet et al. (23) to study the interaction vertex imaging approach for carbon-ion monitoring. However, the application of the PGPL concept was only possible for the data from the setup 1 because the data from setup 2 were too scarce for a meaningful fitting procedure.

### 2.4 Geant4 Improvement

After the comparison between the outcome of the aforementioned models and the experimental data, a systematic study of the possibilities for improvement using each model was carried out. Such a study distinguishes between two cases, one in which built-in options of each model are changed, and the other where changes to the source code are made. It is emphasized that any change in the models is always driven by some physical meaning. If the purpose was otherwise, one could probably apply correction factors to the simulated data. However, this approach may pose additional problems since it may be very difficult to assess the factors for all biologically relevant materials and proton energies. In fact, tuning the free and physically bounded parameters of the Geant4 source code is logical since historically Geant4 was developed for high-energy physics, for which both the projectile energies and targets are significantly different from the ones of medical physics. Therefore, it is expected that hadronic inelastic models and their parameters are optimized mainly for high-energy physics scenarios and that they may be adjusted to yield better accuracy for the application in the study herein. As an example, Dedes et al. (12) found that one of the hard-coded free parameters of the QMD model was optimized for interactions similar to Au + Au. When optimizing that parameter for targets relevant to medical physics, the authors were able to obtain an agreement between experimental and simulated data for prompt-gamma emission yields when considering carbon-ion irradiation.

## 3 Results

### 3.1 Experimental vs. Simulated Data

**Figure 4:**
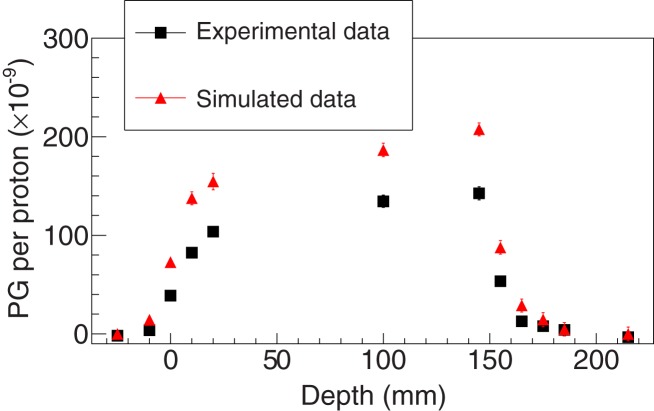
**Experimental and simulated data for setup 2 using the LYSO detector and considering an energy selection of 1 ≤ energy ≤ 7 MeV**. The simulated data were obtained with the BIC model for proton inelastic interactions.

**Figure 5:**
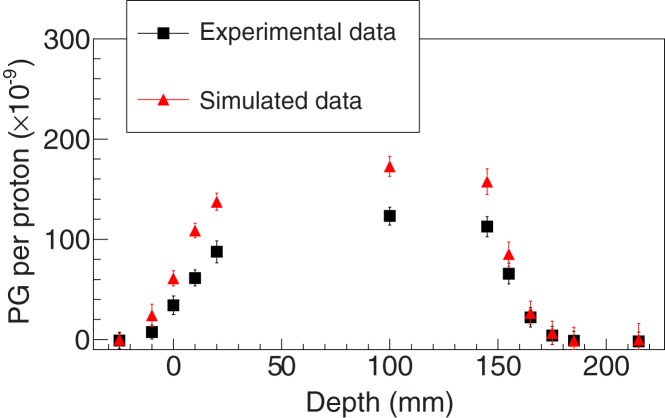
**Experimental and simulated data for setup 2 using the LaBr_3_ detector and considering an energy selection of 1 ≤ energy ≤ 12 MeV**. The simulated data were obtained with the BIC model for proton inelastic interactions.

Figures 3–5 show the experimental and simulated data for both setups and detectors. It can be observed that the simulated data are consistently overestimated with respect to the experimental results. The relative differences are presented in Table 2. Additionally, the fits to retrieve the PGPL are also depicted in Figure 3. The estimated PGPL for the experimental data is 148.3 ± 0.9 mm, while for the simulated case is 148.2 ± 0.8 mm.

**Table 2:** **Average relative difference between experimental and simulated data computed using the data points between 20 and 140 mm**.

	Average relative difference (%)
Setup 1 LYSO	39.9 ± 0.7
Setup 2 LYSO	39.9 ± 0.3
Setup 2 LaBr_3_	41.5 ± 0.6
Average	40.2 ± 0.3

*The average considering the values of the three cases was calculated with the standard weighted least-squares formula (24)*.

It should be noted that the error bars were estimated with the same procedure followed by Pinto et al. (17), in which the statistical uncertainties (1 SD) for each data point and the uncertainties imparted by the background subtraction method are taken into consideration. Due to the nature of the latter, it is not unreasonable to consider that the error bars may be under-/overestimated since it is not possible with the current set of data to estimate accurately the background superimposed with the prompt-gamma signal.

### 3.2 Default Proton Hadronic Inelastic Models

**Figure 6:**
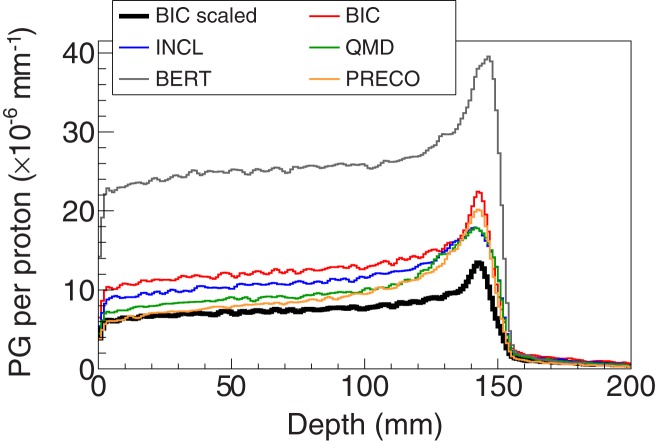
**Longitudinal profiles of the photons escaping the PMMA target having an angular acceptance of ±1.5°**. These profiles were obtained with the default models of Geant4 and the “BIC scaled,” which corresponds to the BIC case scaled down to compensate the estimated overestimation of 40.2% (see Table 2).

**Figure 7:**
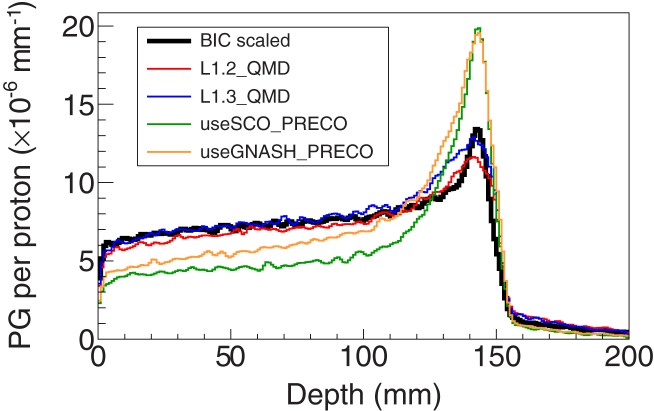
**Longitudinal profiles of the photons escaping the PMMA target having an angular acceptance of ±1.5° using a given built-in option in Geant4 or a change in the source code**. The “BIC scaled” profile refers to the BIC case scaled down to compensate the estimated overestimation of 40.2% (see Table 2). The naming conventions are presented in Table 3.

Figure 6 shows the longitudinal profiles obtained with the default implementation of all applicable Geant4 proton inelastic models along with the reference model scaled down to account for the estimated overestimation. Since the different upper-energy thresholds did not have an impact on the overestimation, the simulated data depicted in Figures 6 and 7 consider events with 1 ≤ energy ≤ 12 MeV.

### 3.3 Improved Proton Hadronic Inelastic Models

Figure 7 depicts the longitudinal profiles obtained after using a given built-in option of Geant4 or making a given change in the source code. The naming used and the different changes are summarized in Table 3.

**Table 3:** **Built-in options (type 1) and source code changes (type 2) tested allowing for a reduction in the prompt-gamma emission yields compatible with the expected experimental data**.

Option	Naming	Model	Type	Geant4 class
Wave packet width equal to 1.2 fm^2^	L1.2_QMD	QMD	2	G4QMDParameters
Wave packet width equal to 1.3 fm^2^	L1.3_QMD	QMD	2	G4QMDParameters
Use of soft cutoff for deexcitation	useSCO_PRECO	PRECO	1	Not applicable
Use of GNASH transitions	useGNASH_PRECO	PRECO	1	Not applicable

*The column “Geant4 class” refers to the class where some source code change was done if applicable*.

## 4 Discussion

The results herein show that Geant4 consistently overestimates the prompt-gamma emission yields for the present case, which is in agreement with the conclusions of previous studies. However, the main difference of this study is the use of all applicable proton inelastic models for the energy regime in medical physics. As already suggested elsewhere (8), Bertini cascade model is the one yielding the worst agreement. This was partially corrected when using the precompound model as the model for the pre-equilibrium stage instead of its own implementation. This profile was not shown because, even after this change, the yields are at the same level as the default binary cascade model. The emission predicted by the default precompound model for shallow depths is accurate but then it increasingly diverges from the expected yields along the depth.

In addition, the PGPL is in excellent agreement between experimental and simulated data for the single case investigated. However, the assessment of the accuracy of simulations in estimating it in clinical conditions can only be performed when a shift of proton range is considered and the subsequent correlation with proton range is determined. Therefore, further studies with increasingly complex phantoms are required to assert such an agreement [for example, studies similar to Priegnitz et al. (25)]. Nonetheless, it indicates that even if Geant4 overestimates the prompt-gamma yields, it still can predict accurately the PGPL for the present case.

Concerning the improvement of Geant4, it is possible to observe that the QMD model using the wave packet width equal to 1.3 fm^2^ yielded the best agreement with the BIC scaled case. This value contrasts with the one proposed by Dedes et al. (12) for carbon-ion irradiation, which was 0.8 fm^2^, but that work dealt with a different projectile and systems with substantial higher energy. The default value for the wave packet width in the QMD model is 2 fm^2^. This indicates that further studies are required to fully assess the most adequate value for this parameter, namely, with other clinically relevant targets and energies. The cases presented with the precompound model show a clear underestimation of the PG yield for most of the proton path. However, it increases distally to values that are similar to the ones obtained without the use of the built-in options (compare the PG profile using the default precompound model in Figure 6 and the ones in Figure 7). Even though no testing was performed to find the reason for this behavior, one can assume that it may be related to the modeling stage after the precompound, i.e., the deexcitation. The lower the proton energy the more likely it is to send the fragments to the deexcitation earlier in the modeling process, as the fragments will have gradually less excitation energy. Although only four changes have been shown, many others were attempted but they yielded either a non-significant or an excessive reduction in the PG emission yields. Usually, the discussion about improving simulations is linked to the improvement of cross sections [e.g., Ref. (15) for the discrete emission]. However, even though the cross section data available should indeed be improved, the type of approach followed herein provides Geant4 users with additional possibilities as they can also improve their outcomes through scientifically sound changes to the default implementation of Geant4, both in terms of the default options chosen by the developers and the free and physically bounded parameters.

It should be noted that accurate cross sections are, in general, important for a better modeling of the prompt-gamma emission. However, most of the applicable models in Geant4 are model-driven and not data-driven; hence, the simulation outcomes are based on sound physical models benchmarked with available experimental data. In the prompt-gamma emission context, this is not true for the discrete emission that relies on tabulated data for the possible nuclear transitions. Therefore, for most cases, only the total inelastic hadronic cross sections are used to then sample an inelastic hadronic interaction. Since the present work addresses the total prompt-gamma emission (continuous and discrete), and it is known that the total inelastic hadronic cross sections in Geant4 are relatively accurate for the present application [e.g., see Ref. (15)], the authors did not consider an in-depth study of the implemented cross section data in Geant4 and their influence in the total prompt-gamma emission.

Regardless of the model and the parameter to optimize for a practical application of Geant4 in the clinical routine of proton therapy and prompt-gamma monitoring, the approach toward the improvement of Geant4 will ultimately depend on the experimental data gathered with different materials and proton energies and the outcomes of Geant4 after those conditions. If a single parameter value yields a good agreement with such data while in accordance with the nuclear physics theory, then it would be straightforward to have it implemented within the models by simply replacing the default value. However, if it is not the case (i.e., dependency with the target nuclei and/or energy), one needs to find the corresponding values strengthened by theoretical developments and, for example, implement a look-up table of parameter values for several material-energy pairs to be used with the models.

## Author Contributions

The contributors listed in the author list meet at least one of the four authorship criteria following International Committee of Medical Journal Editors (ICMJE) recommendations.

## Conflict of Interest Statement

The authors declare that the research was conducted in the absence of any commercial or financial relationships that could be construed as a potential conflict of interest.
